# Cellular Chondroitin Sulfate and the Mucin-like Domain of Viral Glycoprotein C Promote Diffusion of Herpes Simplex Virus 1 While Heparan Sulfate Restricts Mobility

**DOI:** 10.3390/v14081836

**Published:** 2022-08-21

**Authors:** Yara Abidine, Lifeng Liu, Oskar Wallén, Edward Trybala, Sigvard Olofsson, Tomas Bergström, Marta Bally

**Affiliations:** 1 Department of Clinical Microbiology, Umeå University, SE-90185 Umeå, Sweden; 2 Wallenberg Centre for Molecular Medicine, SE-90185 Umeå, Sweden; 3 Department of Infectious Diseases, Institute of Biomedicine, University of Gothenburg, SE-41346 Göteborg, Sweden

**Keywords:** herpesvirus, glycosaminoglycan, mucin-like domain, virus diffusion, glycocalyx, viral O-glycans, single particle tracking, glycocalyx

## Abstract

The diffusion of viruses at the cell membrane is essential to reach a suitable entry site and initiate subsequent internalization. Although many viruses take advantage of glycosaminoglycans (GAG) to bind to the cell surface, little is known about the dynamics of the virus–GAG interactions. Here, single-particle tracking of the initial interaction of individual herpes simplex virus 1 (HSV-1) virions reveals a heterogeneous diffusive behavior, regulated by cell-surface GAGs with two main diffusion types: confined and normal free. This study reports that different GAGs can have competing influences in mediating diffusion on the cells used here: chondroitin sulfate (CS) enhances free diffusion but hinders virus attachment to cell surfaces, while heparan sulfate (HS) promotes virus confinement and increases entry efficiency. In addition, the role that the viral mucin-like domains (MLD) of the HSV-1 glycoprotein C plays in facilitating the diffusion of the virus and accelerating virus penetration into cells is demonstrated. Together, our results shed new light on the mechanisms of GAG-regulated virus diffusion at the cell surface for optimal internalization. These findings may be extendable to other GAG-binding viruses.

## 1. Introduction

The initial recruitment of a virus particle to the cell surface is a complex, dynamic multistep process that requires navigation through the glycocalyx, the sugar coat covering cells, which can exhibit a thickness of up to one micrometer in some tissues [1], to reach the plasma membrane where the virus may further laterally diffuse in search of a suitable point of entry [2,3]. Every event in this process is orchestrated by specific biomolecular interactions between virus and receptors on the cell surface, and each step is an opportunity for the cell to trap the virus in this soft, gel-like meshwork containing multiple possible binding sites. Multivalency, the virus particle’s ability to form multiple biomolecular contacts simultaneously with the cell surface, contributes to the regulation of the avidity and characteristics of virus–membrane interactions [4]. A tight regulation of such interactions is essential: indeed, a too strong binding, due to a high number of receptors with high affinity, could trap the virions at the cell surface or in the glycocalyx and prevent their diffusion towards entry receptors; in this case, the GAG acts as a decoy receptor. On the other hand, a too weak binding may not be sufficient for the recruitment of the particle or to ensure sufficient residence time for entry [3].

Many viruses initiate their recruitment on the host cell by taking advantage of carbohydrates found on the cell surface and in the glycocalyx. In particular, sulfated glycosaminoglycans (GAGs), such as heparan sulfate (HS) and chondroitin sulfate (CS), are ubiquitously exposed as cell-surface polysaccharide chains and exploited by many human viruses, including herpes simplex virus (HSV) types 1 and 2 [5,6], human papillomaviruses [7], respiratory syncytial virus [8], and severe acute respiratory syndrome coronaviruses 1 and 2 [9,10], to bind and accumulate at the surface of a host cell prior to infection [11]. For enveloped viruses, the GAG–virus interactions are mediated by viral glycoproteins found on the viral surface. These glycoprotein–GAG bonds are typically of weak affinity, which could be beneficial for the transport of the virus through the GAG-rich glycocalyx.

While the identification of the key molecular players involved in GAG–virus interactions has long been the focus of investigations [5,12], much less attention has been devoted to the dynamic nature of such interplay, especially during the initial phase of virus recruitment. Specifically, how the pathogens can diffuse through the GAG-rich cellular glycocalyx and the cell membrane to be efficiently directed to their site of entry without being trapped remains unclear. Some sialic-acid-binding viruses, such as influenza viruses, have solved this remarkable problem by expressing a receptor-degrading enzyme (e.g., neuraminidase for Influenza A) in addition to a receptor-binding enzyme (e.g., hemagglutinin for Influenza A) [13,14]. Thus, a delicate interplay between a receptor-binding viral glycoprotein and a receptor-degrading viral enzyme provides a means of locomotion at the cell surface and in the glycocalyx [15,16]. For GAG-binding viruses, no viral GAG-degrading enzyme appears to be involved in the process, suggesting that other molecular mechanisms are at play. One compelling scenario is that, in this case, virus navigation is facilitated by reversible, relatively low-affinity and fast-exchange-rate interactions, permitting a diffusional movement through the formation and dissolution of individual bonds between the multivalent virus and the cell surface. A similar mechanism involving the translocation from one HS-binding site to another has been reported for the diffusion of GAG-binding proteins [17]. Our own data, visualizing the binding of HSV-1 to surface-immobilized GAG molecules, also suggest such a behavior as a possible transport mechanism for viruses [16].

For HSV-1, a prototype herpesvirus of 150–200 nm diameter [18], the virus entry process is exceptionally complex, involving, in a cascade-like manner, a majority of the more than ten glycoprotein types that are available in the viral envelope, especially those designated as gC, gD, gH, gL and gB. HSV-1 infection is initiated by the interaction of virus-associated gC with the outermost GAG layer of the target cell [5,6,16,19,20,21,22,23], primarily via binding to HS and CS via overlapping domains of the viral glycoprotein [6,12,22,23,24,25,26]. Subsequently, viral navigation towards the lower parts of the GAG layer, proximal to the cell membrane, occurs, enabling gC to prime gD for the activation of other envelope glycoproteins that will interact with host target molecules, through binding to 3-O-sulfated HS and/or receptors of protein nature situated close to the plasma membrane of the target cell. The viral-envelope-associated complex gH and gL finally triggers the fusion activity of gB to allow for the entry of HSV-1 into the target cell [27]. The activities of gC in this process differ from those of gD, gH, gL and gB, whose interactions all occur physically close to the cell membrane, while the initial contact mediated by gC targets peripheral GAG structures, which can be situated hundreds of nanometers from the target cell surface. Therefore, one important question pertains to the mechanisms by which viral navigation is carried out, from the initial peripheral gC-GAG contact all the way to the entry receptor where other envelope glycoproteins operate. Although enigmatic and largely unknown, the regulation of viral navigation must be a delicate process, balancing the need for sufficiently strong binding conditions to prevent approaching viruses from bouncing away from the target cell, and weak enough interactions to permit adequate mobility and dynamic behavior in the glycocalyx and at the cell surface.

A lead for solving this problem was provided by our previous work on HSV-1, suggesting that the nature, density, and degree of cell-surface GAGs’ sulfation may contribute to modulating virus–GAG interactions [28]. Indeed, using biomimetic surfaces mimicking the cell-surface glycocalyx, we have shown that HSV particles can diffuse on GAGs, presumably through a receptor exchange mechanism where the formation and dissolution of individual bonds between the viral glycoprotein and neighboring cellular GAGs results in a rolling or hopping behavior; this behavior is influenced by the nature of the GAGs [16,21]. The modulatory role of cellular GAGs on virus interactions at the cell surface is further highlighted in a recent work reporting that the removal of HS at the surface of HSV-infected cells, through an increase in cellular heparanase expression, may facilitate virus particle detachment upon egress and prevent reentry into parental cells [29,30].

In addition to the cellular GAG properties, two lines of evidence suggest that gC itself is not only important in the context of its role as a binder of peripheral GAGs of the target cells but is also engaged in modulating viral navigation to the plasma membrane. First, a wealth of accumulated data from a number of human and animal herpesviruses support the notion that viral glycoproteins decorated with clusters of O-glycans, referred to as mucin-like domains (MLD), are important modulators of virus–GAG interactions during their egress from the parent cell [14,26,28,29,30]. For HSV-1, the MLD located on the viral glycoprotein gC has been shown to balance virus attachment and detachment by regulating the affinity, accessibility, and number of GAG–virion contacts formed at the cell surface, thereby facilitating the release of HSV-1 from the cell surface [20,31]. Secondly, studies on the interactions between purified gC protein molecules and surface-associated GAGs identify the viral MLD itself as a key structure that is able to regulate the number of gC molecules associated to a single GAG chain by one order of magnitude [20]. Together, these observations encourage the hypothesis that the HSV-1 glycoprotein gC is instrumental in promoting successful viral navigation through the glycocalyx and at the cell surface during early interactions with HSV-1 and its target cell.

These hypotheses stem from previous results on biomimetic models mimicking the cell-surface glycocalyx, which suggest that the GAG nature and viral MLD play a role in influencing virus diffusion; however, more direct investigations into the virus behavior at the surface of living cells are needed to delineate the mechanisms of early attachment and viral navigation. Therefore, we followed the GAG-dependent diffusion of HSV-1 at the surface of live cells, with the aim of identifying its modulatory mechanisms. Specifically, we used single particle tracking (SPT) and live cell microscopy to dissect the heterogeneous diffusion behavior of HSV-1 at the cell surface prior to uptake, and further correlated the diffusion behavior with the efficiency of viral entry. Here, we investigate the molecular mechanisms underlying viral diffusion on the cell surface GAGs of Chinese hamster ovary cells (CHO-K1) and human keratinocytes (HaCaT) cells. Although the glycocalyx of these in vitro cells is not expected to exceed a few tens of nm, they represent suitable models to study the GAG-dependent motion of HSV-1 at the cell surface. We report the different roles of cellular HS and CS during the initial steps of the virus interaction with CHO-K1 and HaCaT cells: CS enhances free diffusion while HS promotes virus confinement. Furthermore, we found that gC is a major regulator of viral navigation in the glycocalyx, thanks to its carbohydrate-rich MLD, which facilitates viral diffusion. We suggest that such optimized and fine-tuned interactions are necessary to ensure efficient viral navigation through the glycocalyx to cell membrane sites, where the entry receptors are available for the sequential action of other viral glycoproteins.

## 2. Materials and Methods

### 2.1. Cell Culture and Enzymatic Treatment

Wild-type Chinese Hamster Ovary CHO-K1 cells, heparan sulfate-deficient pgsD-677 (ATCC CRL-2244) cells and glycosaminoglycan-deficient pgsA-745 (ATCC CRL-2241) cells were a gift from N. Arnberg (Umeå University, Sweden). GAG analysis of these mutants reveals that GAG biosynthesis is inhibited in pgsA mutants due to defects in xylosyltransferase [32,33], while pgsD mutants have a N-acetylglucosaminyl- and glucuronosyltransferase defect, which results in heparan sulfate deficiency [22,34]. Wild-type human keratinocytes (HaCaT) cells were a gift from M. Evander (Umeå University, Sweden). CHO cells were cultured in Ham’s F-12 nutrient mixture (Gibco 21700-075, Fischer scientific, Göteborg, Sweden) supplemented with 5% fetal bovine serum (FBS, SV30160.03, Cytiva, Uppsala, Sweden), penicillin 100 U/mL and streptomycin 100 ug/mL (Gibco 15140-122). HaCaT cells were cultured in DMEM medium (D5648-10L, Merck, Solna, Sweden) supplemented with 5% fetal bovine serum, penicillin 100 U/mL and streptomycin 100 μg/mL. All cells were cultured at 37 °C in a 5% CO_2_ incubator. For live cell SPT and immunofluorescence microscopy, cells were seeded 24 h before experiments on 4-chamber 20 mm glass-bottom dish (220.120.022, IBL GmbH, Gerasdorf bei Wien, Austria) to reach a density of 2.10^5^ cells/well on the day of imaging. Before all experiments, cells were washed with serum-free medium once gently and on the side of the dish to avoid damaging the cell surface. The experiment was carried out in serum-free MEM or DMEM supplemented with 20 mM 4-(2-Hydroxyethyl) piperazine-1-ethanesulfonic acid, N-(2-Hydroxyethyl) piperazine-N′-(2-ethanesulfonic acid). The enzymatic treatment with chondroitinase ABC (from Proteus vulgaris, C3667, Merck, Solna, Sweden and heparinase I and III (from Flavobacterium heparinum, H2519 and H8891, Merck, Solna, Sweden) was carried out by incubating cell monolayers with 1 U/mL of the enzymes for 1 h at 37 °C. All enzymes were reconstituted at 50 U/mL stock concentrations in buffers, according to the enzyme manufacturer instructions: 50 mM Tris-HCL, pH = 8. 60 mM sodium acetate and 0.02% BSA for chondroitinase ABC, and 20 mM Tris-HCL, pH = 7.5, 50 mM NaCl, 4 mM CaCl_2_ and 0.01% BSA for heparinase I and III. Immediately prior to treatment, the stocked enzymes were diluted in a warm digestion buffer (HAM F12 or DMEM medium and 0.2% bovine serum albumin (Fraction V Ref 10735094001, Merck, Solna, Sweden), pH = 8 for chondroitinase ABC and pH = 7.5 for heparinase I and III). An immunofluorescence assay showed that enzymatic treatment of CS was effective and reduced CS intensity levels (Figure S3A). For the native and mutant virus strains’ purification, African green monkey kidney cells (GMK AH1) were used and cultured in Eagle’s minimum essential medium (EMEM) supplemented with 2.5% fetal bovine serum and 1% penicillin/streptomycin.

### 2.2. Viruses and Fluorescence Labelling

Herpes simplex virus 1 (HSV-1) strains KOS (VR-1493; ATCC, Manassas, VA, USA) and the mutant variant KOS-gCΔmuc [20,35], lacking the mucin-like domain (amino acids 33-116) in the glycoprotein C, were purified from infectious culture media of GMK AH-1 cells using a three-step discontinuous sucrose gradients [36]. The virus was produced using MOI of 0.01 on GMK AH-1 cells and the virus inoculum was collected 72 h after infection. All the purified virus suspensions were quantified using viral plaque titration assays on GMK cells. Viruses were labelled immediately prior to live cell microscopy with fluorescent lipophilic dye 3,3′-Dioctadecyl-5,5′-Di(4-Sulfophenyl) Oxacarbocyanine, Sodium Salt (SP-DIOC18 (3)) (D7778, Invitrogen, Burlington, Canada) and incubated for 1 h at 4 °C. Unbound dye was removed via buffer exchange into phosphate-buffered saline (PBS) using gel filtration columns (Amersham MicroSpin S-200 HR columns, 27-5120-01, Cytiva, Uppsala, Sweden) [16,21]. Immediately before experiments, viral aggregates were removed by spinning down the virus solution and diluted in HAM medium or DMEM without phenol red (11570406, Fischer scientific, Göteborg, Sweden). Losses of labeled viral particles due to buffer exchange were estimated to be 60% using Förster Resonance Energy Transfer (FRET)-based assay [21], and were taken into account when calculating the multiplicity of infection.

### 2.3. Epi-Fluorescence Microscopy

Live-cell and immunofluorescence imaging was carried out using a Nikon Eclipse Ti2-E inverted microscope equipped with a Spectra III solid-state light source, a Prime 95B sCMOS camera (Teledyne Photometrics, Birmingham, UK) and a multibandpass filter cube 86012v2 DAPI/FITC/TxRed/Cy5 (Nikon Corporation, Melville, USA). The images were recorded using a 60× oil immersion objective (NA = 1.49), and LED light source combined with a 488 nm filter and NIS software. Immediately prior to imaging, diluted SP-DIO-labeled viruses were added at MOI = 100 in the dish after the medium was removed from live cells. The viruses and cells were then immediately imaged, without rinsing, to ensure that landing viruses were detected. Movies were recorded at an acquisition rate of 25 frames per second for 2 min. The timeframe of 2 min was chosen as the optimal time to ensure that enough viruses landed on the cells without recording their internalization, while also avoiding bleaching the fluorescent particles. During live-cell imaging, the cells were kept in an on-stage incubator (37 °C, 5% CO_2_).

### 2.4. Immunofluorescence Staining and Imaging

Immunofluorescence staining (IF) was used to verify the presence of GAGs on the different CHO-K1 and HaCaT cells following every SPT experiment to ensure the levels of HS and CS were stable and repeatable between each experiment. To do this, IF was performed in one of the wells of the 4-wells chamber adjoining the one used for SPT, thereby ensuring a verification of the amount of HS and CS at the cell surface for each condition used. Cell monolayers were fixed with 4% paraformaldehyde in medium for 15 min. The fixed cells were then incubated with mouse monoclonal anti-HS antibody (clone 10E4, AMSBIO 370255-S) and mouse monoclonal anti-CS (CS-56, C8035, Merck, Solna, Sweden) at 1:200 dilution in binding buffer (0.5% bovine serum albumin, 2 mM ethylenediaminetetraacetic acid, Ph = 8.5) at 4 °C overnight. This was followed by 2 h incubation at room temperature with the secondary antibody AlexaFluor 488-conjugated goat anti-mouse IgG/IgM (H+L) (A-10680, Invitrogen, Burlington, Canada) at 1:500 dilution in binding buffer. Between each step, cells were rinsed 3 times with cold PBS. Finally, the cells’ nuclei were stained with Hoescht 33342 (ab228551, Abcam, Cambridge, UK) at 1 µg/mL for 15 min at RT. The cells were kept in PBS at 4 °C until imaging. A control was also performed using the secondary antibody only, and used to correct the fluorescence intensity. For each dish, a minimum of 7 images were acquired using epifluorescence microscopy with a 60× oil immersion objective and 405–488 nm filters and differential interference contrast (DIC). For HaCaT cells, the enzyme verification was carried out in a separate experiment where the cells were treated with heparinase I and III (1 U/mL) and chondroitinase ABC (1 U/mL), followed by the same staining protocol as described above. The imaging was completed using a 40× objective.

Analysis of the images was then performed using a homemade ImageJ script. For each image, the fluorescence intensity per pixel was measured and the background intensity was subtracted using three background regions. This value was then normalized by the secondary antibody control fluorescence intensity. The number of cells per image were measured using DAPI channel and the mean fluorescence intensity per cell was calculated.

### 2.5. Single Particle Tracking (SPT)

Recorded timelapses were processed and analyzed using Trackmate [37] and matlab DC-MSS [38]. First, a Fiji [39] built-in macro subtracted the background noise of the movie by correcting the unevenness with a rolling ball of 50 combined with noise filtering using despeckle. The virus particles were then detected using the Laplacian Gaussian filter with sub-pixel localization in the Trackmate plugin, followed by building the trajectories using a linking frame-to-frame displacement of 5 μm and a maximum gap of 5 μm and 20 frames. Only landing virus particles were accounted for in the analysis by ignoring all tracks that started before frame 10. Aggregates from the tracking analysis were ignored. Then, trajectories longer than 180 frames were chosen for segmentation and classified in matlab using a built-in script and DC-MSS. The diffusion was classified using the moment scaling spectrum (MSS) that uses high order moments of displacement and the slope of the MSS reflects the motion type: a slope of 0.5 implies free normal diffusion, a slope between 0 and 0.5 yielded a confined motion, a slope >0.5 represented superdiffusive directed motion and finally a slope ≤0 gave the immobile particles. Segmentation of the trajectories was dependent on the type of motion, using a rolling-window of 21 frames where consecutives frames with the same classification were grouped to yield segments of different diffusion types. The frame window was chosen as the minimum segment length for the initial classification to avoid classification errors [38]; however, after being pooled depending on their diffusion type, the segments could end up being longer than 21 frames (as represented in Figure S4). For each segment, diffusion properties such as the duration, diffusion coefficient (*D*) and confinement radius (*R_C_*) were extracted, as detailed in Vegas et. al. [38] The shortest segment was 0.8 s, representing 20 frames, and ensured that enough frames were used for the MSS analysis and subsequent classification of the diffusive behavior. Since segmentation was performed using a moving window, each segment duration was not a predetermined parameter and depended solely on the diffusive behavior. Using a homemade script, the output data used in this study were then computed: classified segments from all experiments in a given condition were pooled together, and the probability of one segment being immobile, confined or in free motion was deduced by dividing the sum of all segments in one motion type by the number of all segments. Similarly, the time spent in one motion type was calculated as the ratio of the sum of time spent in one motion type to the total time spent by all segments in all motion types. The distributions of the diffusion coefficient of each segment were plotted and showed a multimodal distribution, with two populations, herein referred to as the “slow” and “fast” segments with a cutoff that was arbitrarily set at 10^−2^ μm^2^.s^−1^. All graphs were plotted in Graphpad.

### 2.6. Quantification of Virus Binding

The amount of virus attached to the cell surface was quantified by qPCR. Cells were seeded at about 80% confluency in 12-well plates. Virus of 200 PFUs diluted in 150 µL infection medium (DMEM, 1%FBS, 20 mM HEPES, and 1% Penicilline and Streptomycin) was added to each well. Virus-binding synchronization was conducted on ice while shaking the plate every 10min for 1h, followed by two cold washes with PBS to remove unbound viruses. After attachment, the viruses and the cells were harvested for DNA extraction according to the manufacture’s instruction (Invisob Spin Virus DNA Mini Kit, IBL GmbH, Gerasdorf bei Wien, Austria). Extracted DNA was quantified for HSV1 copies with primers targeting the US5 (unique short) gene [40]. Absolute quantities of HSV1 copies were obtained by a standard curve, established using pI18-HSV1-US5 as the template. The two primers used for qPCR were as follows: HSV1-US5-F, (5′-GGCCTGGCTATCCGGAGA-3′); HSV1-US5-R (5′-GCGCAGAGACATCGCGA-3′). The qPCR probe was (5′-6FAM-CAGCACACGACTTGGCGTTCTGTGT-Dark Quencher-3′). The qPCR program was run at 95 °C for 3 min; 40 cycles (95 °C, 15 s; 60 °C, 30 s). Since the virus concentration (200 PFUs) was too low for stable detection via qPCR, binding quantification was achieved after increasing the amounts of virus to 5000 PFUs and 25,000 PFUs. The quantification of different PFUs as inputs by qPCR revealed an almost perfect linear regression (Supplementary Figure S8), confirming that this approach was viable. The binding data for the enzymatically treated conditions and for KOS and KOS-gCΔmuc were then normalized to the control.

### 2.7. Viral Entry Efficiency Assay

All entry experiments were conducted in 12-well plates with 200 PFUs as inputs. Prior to entry at 37 °C, virus binding was performed as described above. After two cold PBS washes on ice, the viruses attached to the cell surface were either inactivated immediately (t = 0 min) by low pH buffer (pH = 3, 40 mM citric acid, 10 mM KCl, 135 mM NaCl) [41,42] or shifted to 37 °C for active entry for selected periods of time (t = 10, 20, 30, 60 and 90 min) before low PH buffer inactivation. Low PH buffer inactivation was carried out by adding 350 µL/well of low pH buffer for 2 min on ice with continuous shaking every 20–30 s, followed by two PBS washes. After inactivation of the virus, the cells were covered by infection medium and kept at 37 °C until the last point of virus inactivation. Thereafter, infection medium was removed, and cells were covered by 1% agarose in DMEM (5% FBS) for another 3 days in culture until obvious plaque formation. A low MOI was chosen to ensure a clear readout of plaque formation. Cells were then fixed with 4% formaldehyde (in PBS) and stained with crystal violet (1% crystal violet in 20% ethanol solution). The plaque was then counted for each condition. The quantification of the virus entry efficiency was carried out by dividing the number of plaques by their corresponding binding factors.

### 2.8. Statistical Analysis

All statistical analyses were performed with GraphPad by using a Welch *t*-test and Student’s *t*-test. Statistical relevance was reached for *p* ≤ 0.05 (*), *p* ≤ 0.01 (**), *p* ≤ 0.001 (***) and *p* ≤ 0.0001 (****) and *p* > 0.05 (ns) was considered non-significant. Means are presented with the standard error of the mean (SEM).

## 3. Results

### 3.1. Single-Particle Tracking of Individual HSV-1 Virions Reveals That Viruses Undergo Both Free and Confined Motion upon Landing on the Cell

The dynamics of the initial phase of virus particles binding to the cell were investigated via SPT analysis of labelled viruses landing on the cell during the experiment, as seen in Movie S1A (Supporting Material). Labelled viruses were added immediately prior to imaging and kept without rinsing for the duration of the tracking experiment. A maximal track duration of 2 min further ensured that the viruses were not internalized during the tracking process. Indeed, studies on CHO-K1 and HaCaT cells revealed very little internalization within 2 min [42,43], which was also in line with our observation that less than 0.3% of cell-associated HSV-1 particle (KOS) were internalized into CHO cells within 5 min of inoculation (Figure S1A).

**Figure 1:**
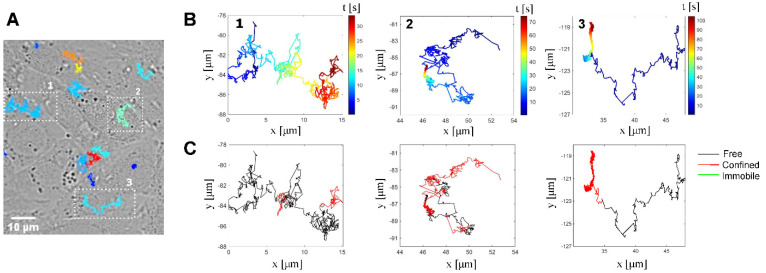
Single particle tracking. (**A**) Overlay of a differential interference contrast (DIC) image of CHO cells and fluorescence image of SP-DIO-labeled HSV-1 particles captured immediately before recording the virus movement (corresponding Movie S1A with maximum projection of a 120 s time-lapse sequence acquired at 25 fps). (**B**) Full tracks of three representative tracks (1 – 2 – 3) of viruses landing on the cell surface extracted with Trackmate. The associated movie is shown in Movie S1E. (**C**) Segmentation of the representative tracks shown in B and classification of segments using moment scaling spectrum (black: Brownian free motion—red: restricted confined motion—green: immobile).

Upon landing on the cell, viruses were found to exhibit prominent mobility. Indeed, virus particles attaching to the cell surface showed lateral diffusion following the initial binding (Movie S1D), as can be seen in the representative tracks shown in Figure 1A,B. Tracks were segmented using a moving window where each segment was classified into four different types of motion—immobile, confined, free or directed motion—via the moment scaling spectrum (MSS) analysis of the displacement [38]. Typically, particles on the cell surface can either be anchored to immobile membrane components [44] or diffuse using various motion types depending on their interaction with their membrane-bound receptors [45]: they can move in a so-called free or Brownian motion, which corresponds to the random walk of a particle, driven by energy fluctuations in the surrounding environment [46], or they can diffuse in a confined motion, which is defined by movements in a restricted area due; for example, to interactions with the receptors that are linked to the underlying cortical actin network [47] or compartmentalized in lipid domains [48]. Finally, they can undergo a directional movement mediated by the cytoskeleton [49]. Here, the virions’ diffusion behavior was heterogeneous in the sense that the virus particle switched several times between immobile (green), confined (red) and free (black) motion during the experiment (Figure 1C and Figure S2A). Only ~1% of the segments exhibited a directed motion behavior in the first 2 min of the virus–cell interaction; this type of motion was, therefore, excluded from the further data analysis presented below.

Hence, our method combining SPT and segmentation analysis was found to be powerful when studying the initial diffusion behavior of HSV-1 at the cell surface. After highlighting the existence of virus mobility upon landing on the cell surface, efforts were directed to using our experimental strategy to investigate how virus navigation is regulated by different GAG components in the glycocalyx.

### 3.2. CS and HS Differentially Regulate Diffusion of HSV-1 upon Landing on the Cell

Our first goal was to investigate the influence of the two predominant GAGs CS and HS on virus diffusion in the glycocalyx. We used CHO-K1 cells, herein referred to as wt CHO cells, to address this aspect. These cells are an ideal model to investigate the effect of GAGs on initial binding while excluding the contribution of other receptors; indeed they lack the specific entry receptors for HSV-1 [22,50,51] but express GAGs CS (mostly CS-A and CS-B) and HS [52]. Moreover, mutant cell lines lacking HS (pgsD-677, here referred to as CHO-ΔHS) and all GAGs (pgsA-745, CHO-ΔGAG) were readily accessible. PgsD-677 mutants have a defect in their GlcNAc- and GlcA-transferase, which inhibits HS biosynthesis [22,34] while pgsA-745 is defective in xylosyltransferase, which inhibits all GAG synthesis [32]. Accordingly, no HSV-1 binding is expected to occur on the CHO-ΔGAG cells (as confirmed experimentally, as in Figure S9), while binding to CHO-ΔHS is exclusively mediated by CS, since there are no other binding receptors for HSV-1 on CHO cells. Since CS deficient CHO cells were not available, CS was enzymatically removed from the cell surface of wt CHO cells by treating them with chondroitinase ABC (ChABC), an enzyme which cleaves types A, B and C of CS [53,54], yielding CHO-ΔCS cells. After ChABC treatment, the CS levels on the cell surface were reduced to undetectable levels by immunofluorescence (Figure S3A). Immunofluorescence further revealed that the wt CHO and CHO-ΔHS cells used here had similar CS expression levels, while CHO-ΔHS yielded, as expected, a significantly lower HS expression than wt CHO (Figure S3B). Our observation that CHO-ΔHS have similar CS levels to CHO cells (Figure S3A) contradicts reports indicating that pgsD-677 mutants exhibit a change in CS biosynthesis, with a CS accumulation that is three times higher than found in wild-type cells [22]. While we did not observe such an increase in CS on CHO-ΔHS using our IF validation method, we cannot exclude the possibility that CS levels are somewhat increased in these cells. However, this would not affect our interpretation, since an increase in the CS levels of the mutants would accentuate the role of CS in this process.

We first confirmed that GAGs play an essential role in the recruitment of virus particles to the surface of CHO cells. While viruses are readily attached to the surface of wt CHO cells immediately upon adding the virus inoculum, very little bound to the CHO-ΔGAG cells: the number of particles that were bound per cell after 10 min was less than 4% of the amount of particles per cell on wt CHO (2.6 ± 0.22 virus/cell for wt CHO while only 0.10 ± 0.03 viruses/cells were recorded for CHO-ΔGAG, Figure S9). As SPT experiments were performed up to 2 min after the virus particle landed at the cell surface, these results further highlighted that virus–GAG interactions were primarily investigated in our experiments.

A close inspection of the time-lapse movies for the wt CHO, CHO-ΔHS and CHO-ΔCS immediately revealed that the dynamic behavior of the viruses at the cell surface was distinct for the different conditions (Movie S1A–C): at the surface of wt CHO, the viruses diffused in the medium before attaching to the cell, followed by the lateral displacement of the cell-bound viruses. This behavior is distinct for CHO-ΔHS cells where a large proportion of the particles tended to linger at the cell surface before firmly attaching. In contrast, the virus particles were found to bind immediately on the CHO-ΔCS cells and only exhibited a limited displacement at the cell surface.

**Figure 2:**
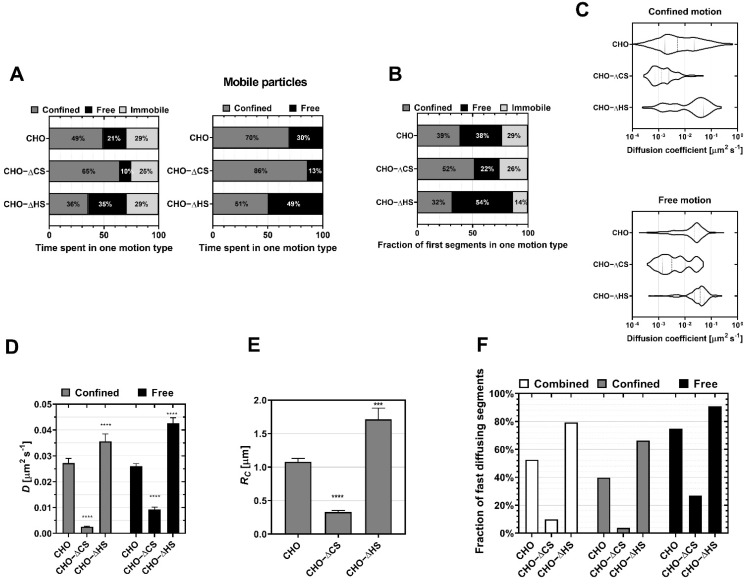
Influence of the nature of the glycosaminoglycans on the initial diffusion of HSV-1 at the surface of non-susceptible CHO-K1 cells. HSV-1 (KOS) diffusion is recorded on CHO-K1 cells expressing both heparan sulfate (HS) and chondroitin sulfate (CS) (CHO), CHO treated with ChABC (CHO-ΔCS) and pgsD-677 cells lacking HS (CHO-ΔHS). (**A**) The fraction of the total time spent by KOS particles in one of the motion types. Left: confined (grey), free (black) and immobile (light gray); Right: mobile particles only (confined (grey) and free (black)). (**B**) The fraction of first segments in one of the three motion types. (**C**) Distributions of the diffusion coefficients plotted in violin plots show two populations: slow and fast particles, classified according to a cutoff of 10^−2^ μm^2^.s^−1^. (**D**) The mean of the diffusion coefficient *D* of the full distribution presented in (**C**). (**E**) The confinement radius *R_C_* of the confined motion for the different cases. (**F**) The fraction of particles exhibiting fast diffusion are presented for the confined and free diffusion combined (white), and for each separated motion type. The total number of viruses used here is 644, 204 and 176 for CHO, CHO-ΔCS, CHO-ΔHS, respectively. The data were acquired in at least three independent experiments. Asterisks denote a significant difference by Welch *t*-test: *** *p* ≤ 0.001 and **** *p* ≤ 0.0001 relative to control. The percentage of fraction (**A**,**B**) is obtained from counting, so no statistical analysis is provided.

Further quantitative insights into the dynamics of virus particles at the cell surface, as a function of its GAG profile, were acquired by analyzing the virus’ diffusive behavior immediately after binding to the different cells. The extracted virus trajectories were segmented by classification in one of the three motion types (immobile, confined, or free), with an average segment length ranging between 3 and 9 s (Figure S4A). For all conditions, the viruses spent most of their diffusion time in a mobile state, and the amount of time spent diffusing was similar for all cases (between 71% and 75% of the time spent in mobile state) (Figure 2A, left). Mobile HSV-1 viruses spent different time fractions in either confined or free motion, depending on the cellular GAG composition (Figure 2A, right), suggesting that the nature of GAGs primarily plays a role in determining the type of mobile motion that is observed. Indeed, the removal of CS (CHO-ΔCS) led to a decrease in the time the virus spent in free motion (13% of the time was spent diffusing freely on CHO-ΔCS, as compared to 30% on wt-CHO); viruses on CHO-ΔHS cells showed the opposite trend, with an increase in time spent in free motion as compared to wt-CHO (49% of the time spent in free motion) (Figure 2A, right). It is worth noting that while the fraction of time spent in each motion type was different for each investigated case, the switch rate between confined and free types was comparable in all cases (Figure S2A). Taken together, these results suggest that CS and HS regulate the diffusive behavior of the initial virus–cell interaction in opposite ways: the presence of CS enhances the free motion of the virus while HS makes it more restricted.

Diffusion analysis extracts the diffusion coefficient *D*, which represents how fast a particle can diffuse through a cross-section. In the case of the confined motion, an area of confinement can be defined as a circle of radius *R_C_* in which the particle diffuses. The distributions of diffusion coefficient for the confined and free motion types (Figure 2C) revealed that the diffusive behavior of HSV-1 on CHO cells was characterized by two distinct populations, with diffusion coefficients each spanning over two orders of magnitude; these two populations are herein referred to as slow and fast populations, respectively, with the cutoff arbitrarily set to 10^−2^ μm^2^.s^−1^. Visually, one can note that for wt CHO, confined motion is characterized by two predominant peaks, while most particles were assigned to the fast population for free motion. This is quantitatively confirmed by extracting the fraction of fast-diffusing particles (Figure 2F) showing that, when both motion types were combined, 53% of the viruses’ segments exhibited fast diffusion (Figure 2F, white). In the absence of CS (CHO-ΔCS), a clear shift in the distribution in favor of the slow population was observed (Figure 2C), with less than 10% of segments exhibiting fast diffusion (Figure 2F, white). In contrast, particles on CHO-ΔHS were shifted to the fast population, with 80% of segments assigned to fast diffusion (Figure 2F, white). Interestingly, qualitatively summing up the CHO-ΔCS and CHO-ΔHS distributions led to a similar distribution to the one observed for the wt CHO (Figure 2C), providing further evidence that HS and CS determine the overall virus diffusion behavior on wt CHO cells. In addition, the type of GAGs exposed on the cell surface was found to influence the average diffusion coefficient *D* (Figure 2D) and the confinement radius *R_C_* (Figure 2E), where *D*_confined_, *D*_free_ and *R_C_* all significantly decreased on CHO-ΔCS, while they all increased on CHO-ΔHS. Taken together, these results indicate that CS makes the virus diffuse faster with a higher area of exploration, while HS contributes to slowing it down, independent of the motion type.

The first segment of each track was also analyzed to directly characterize the behavior of the virus upon landing on the cell surface (Figure 2B and Figure S5). The probability of the virus exhibiting confined or free behavior during the first segment of the trajectory followed the same trend as for all segments, although a higher fraction of the first segments was in free motion for all conditions (Figure 2B). Interestingly, while the first segment lasted between 6 and 9 s in most cases (Figure S5A), the duration of the confined segments in CHO-ΔHS and free segments in CHO-ΔCS decreased compared to CHO, in line with the general idea that CS promotes free diffusion while HS favored confined motion. All other parameters describing the diffusion behavior of the first segment (*D*, *R_C_* and fraction of fast-diffusing particles (Figure S5D–F)) followed the same trend as when all segments were combined, with overall higher diffusion coefficient values. These findings show that, upon landing, the virus preferentially began its navigation at the cell surface with a fast free motion.

Similar tracking experiments were performed using susceptible human keratinocytes HaCaT cells (Figure S6), which express both HS and CS (Figure S6B). HaCaT cells express entry receptors and can more accurately replicate the virus’ in vivo infection process [55,56]. To investigate the role that GAGs plays in virus diffusion in these cells, CS and HS were enzymatically removed from the cell surface by treating them with chondroitinase ABC (ChABC), a mixture of heparinase I and III (Hep), or with a mixture of all enzymes (HepChABC). Immunofluorescence validation of the enzymatic treatments shows that not all CS was removed from HaCaT (Figure S6B-left), suggesting that either digestion with ChABC is incomplete, or other CS types are still present on cells such as CS-D or CS-E (the CS antibody used here, CS-56, targets types A, C and D, but interacts very weakly with types B and E [57]). However, it has been shown that the disaccharide composition of CS chains from HaCaT contains a very low quantity of types D and E as compared to types A and C [58]; thus, it is not likely that HSV-1 was interacting with GAGs other than HS in the ChABC condition. However, Hep treatment decreased HS levels without removing it completely, a residual HS post-treatment effect that has been observed in other studies [59]. This suggests that the Hep condition contains all types of CS as well as some residual HS; this needs to be considered in the interpretation of the diffusion results. Nevertheless, similar qualitative trends were obtained, although the effect of each GAG type on diffusion was less pronounced (Figure S6C–F) than it was on the wt CHO cells. Specifically, the removal of HS via heparinase I and III treatment (Hep) increased the amount of time spent in free diffusion (Figure S6C), as observed with CHO-ΔHS (Figure 2A), confirming HS’ role in promoting confined behavior. However, this did not increase the diffusion coefficient, suggesting that residual HS could have affected the speed of diffusion. However, the removal of CS did not modify the time spent in confined or free motion types, suggesting that the role of the remaining CS-D and E might be more important than expected for the diffusion of HSV-1, even if they are only present in small amounts. Nevertheless, the diffusion coefficient of the confined motion decreased (Figure S6E), as observed on CHO cells, implying that the role of HS is important for the speed of the virus’ diffusion on HaCaT.

In summary, the tracking results highlight how the dynamics of the initial binding of the virus to the glycocalyx and its navigation at the cell surface are affected by structural differences between GAG types. In this context, it appears that CS is essential to ensuring virus mobility, while HS slows down and confines the particle motion. One possibility is that this differential diffusive behavior reflects a strategy devised by virus evolution to successfully navigate the glycocalyx, which would otherwise represent a limitation to viral entry.

### 3.3. HS and CS Differentially Influence the Attachment and Entry Efficiency of HSV-1

**Figure 3:**
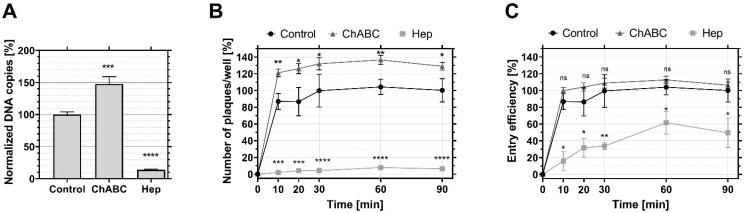
Binding and entry efficiency of HSV-1 in HaCaT cells. HSV-1 (KOS) binding and entry is recorded on wild-type HaCaT cells (Control), HaCaT cells treated with Chondroitinase ABC (ChABC) and HaCaT cells treated with heparinase I and III (Hep). (**A**) Number of virus particles bound to the surface of HaCaT cells after 1h incubation on ice. For quantification, the particles were detached by lysing the cell, and the genomic DNA quantified by qPCR. The data for the control group were normalized to 100. n = 12 for each condition. (**B**) Number of plaques formed over time reflecting the amount of virus internalized into the cells for the control (black circle), chondroitinase ABC-treated cells (ChABC, grey triangle) and heparinase-treated cells (Hep, light gray square). The data were normalized to the average control value at 90 min. (**C**) Entry efficiency defined as the number of viruses internalized into the cell (as measured in (**B**)) divided by the number of virus particles bound to the cell surface (as measured in (**A**)). The data were normalized to the average of control value at 90 min. In (**B**,**C**), each condition has 4 repeated measurements. A Student’s *t*-test was used to assess the significance, ns = not significant, * *p* ≤ 0.05, ** *p* ≤ 0.01, *** *p* ≤ 0.001 and **** *p* ≤ 0.0001 relative to the control HaCaT cells.

To further determine the relevance of the specific mechanisms for HSV-1 navigation through the glycocalyx in the context of the virus’ ability to initiate a productive infection, we quantified the virus entry efficiency, i.e., the percentage of HSV-1 internalized in permissive HaCaT cells prior to and post enzymatic treatments of HS and CS (Figure 3). Firstly, we found that virus binding was significantly reduced after enzymatic treatment with heparinase I and III (Hep) by a factor 8 (Figure 3A), in line with the notion that HS plays a key role in recruiting the virus at the cell membrane. Additionally, treatment with chondroitinase ABC (ChABC) led to a ~20% increase in virus attachment (Figure 3A), indicating that, on the HaCaT cells, CS indeed reduces viral access to other receptors, including HS, which are presumably located closer to the cell membrane [60]. This observation, in line with the fact that the interaction between the virus and CS is generally weaker than that with HS [23], agrees with the notion that apical CS, which is usually longer than basolateral CS [61], on the one hand, enriches the virus at the periphery of the cell but, on the other hand, can constitute a hindrance that needs to be overcome for the virus to reach its entry point at the cell surface.

Secondly, the quantification of HSV-1 entry over time (Figure 3B) showed that the absence of CS promoted HS’s known ability to facilitate viral entry. Thus, the entry efficiency (Figure 3C) after 90 min was reduced by a factor of 2 after treatment with heparinase I and III as compared to the untreated control. Here, 87% of the viruses successfully entered cells within 10 min when HS was present (Figure 3C), while only 16% of the viruses entered in the absence of HS. This confirms the previous idea that HS is not only important in the context of accumulating virus particles at the cell surface, but also plays a key role as a primer for efficient entry [62]. However, the presence of CS did optimize virus recruitment without improving entry efficiency, suggesting that the role of CS is important at the beginning of the interaction between virus and cell.

In summary, these findings support our hypothesis that the virus takes advantage of the different biophysical properties of cell-surface GAGs, HS and CS in this case, to optimize its navigation through the glycocalyx and its subsequent entry. Indeed, in view of a likely stratified distribution of GAGs in the glycocalyx, one possibility is that the differences in the virus’ binding properties to HS and CS, and the resulting differences in the motion behavior, are designed to facilitate transport of the virus from the distal, possibly CS-rich glycocalyx to the proximal HS rich domain. Our experimental data are compatible with the concept that a weak and dynamic interaction with CS is essential in ensuring transfer to the proximal HS, which primes for virus uptake, without trapping in the outer layers of the cell’s sugar coat.

### 3.4. The Mucin-like Domain (MLD) of Viral Glycoprotein C Promotes Virus Diffusion

Most, if not all, of the initial contacts between HSV-1 and target cell-surface peripheral GAGs are mediated by the HSV-1 glycoprotein gC [20,23,26,31,55,63,64,65], a glycoprotein equipped with a prominent MLD that is decorated with a large number of clustered O-linked glycans. There is convincing evidence that the MLD on gC regulates several aspects of gC’s interactions with GAGs on the target cell, including binding affinity, dynamics and stoichiometry [20,55,63], while facilitating mobility on CS monolayers [20]. Therefore, it appeared important to investigate the influence of the gC MLD of HSV-1 on the virus’ diffusion behavior on the surface of live cells, with the aim of exploring its possible significance in the context of viral navigation through the glycocalyx. To do his, SPT experiments were also carried out, using a HSV-1 mutant lacking the MLD, but containing the GAG-binding site on gC [23,26,66] (KOS-gCΔmuc) (Movie S1D–E) with human keratinocytes (HaCaT cells) as a cell model system. The HaCaT cell line was proposed to accurately mimic in vivo infection of the mucosal epithelium [55,56], making it an ideal model to study the MLD’s effects on the interaction with the glycocalyx.

**Figure 4:**
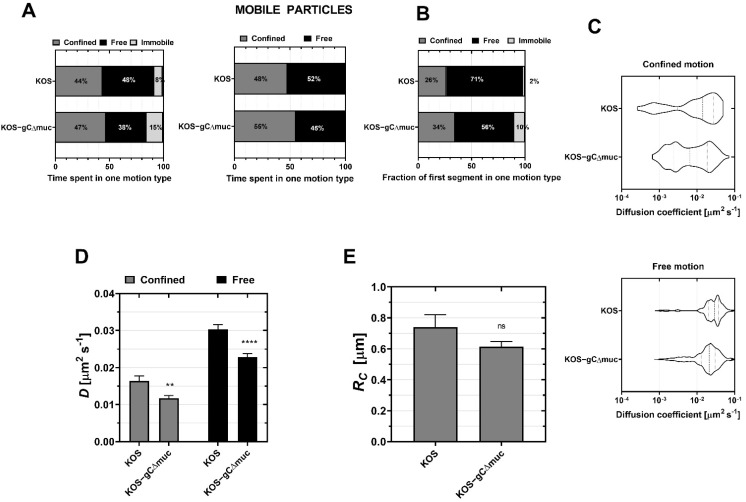
Influence of the mucin-like domain (MLD) of the glycoprotein gC on the interaction of HSV-1 with the HaCaT cell surface upon landing. The diffusion of HSV-1 (KOS strain) and the mutant HSV-1 lacking the MLD (KOS-gCΔmuc) are recorded on HaCaT cells. (**A**) Left: The fraction of the total time spent by KOS and KOS-gCΔmuc in one of the motion types: confined (grey), free (black) and immobile (light gray). Right: The fraction of the total time spent by the two virus strains in the one of the mobile motions: confined (grey) and free (black) (**B**) The fraction of first segments in one of the three motion types. (**C**) Distributions of the diffusion coefficients plotted in violin plots. (**D**) The mean diffusion coefficients *D* of the confined and free motions were calculated for KOS and KOS-gCΔmuc. (**E**) The confinement radius *R_C_* of the confined motion. The total number of landing viruses recorded here is 80 and 201 for KOS and KOS-gCΔmuc, respectively. Asterisks denote a significant difference by Welch *t*-test: ns = not significant, ** *p* ≤ 0.01 and **** *p* ≤ 0.0001 relative to control. The fraction percentage (**A**,**B**) is obtained from counting, so no statistical analysis is provided.

SPT on HaCaT revealed that deletion of the MLD reduced the mobility of the particles (Figure 4A, left) with 15% of the mutant viruses being immobile compared to 7% for the wt KOS. Additionally, the distribution of the diffusion coefficients (Figure 4C) revealed a higher fraction of fast segments for the wt virus. Accordingly, deletion of the MLD significantly decreased the mean *D* in both confined and free motions (Figure 4D) while *Rc* did not significantly change (Figure 4E). Both observations suggest that the MLD is important in ensuring rapid diffusion of the virus at the cell surface. Moreover, deletion of the MLD slightly reduced the time spent in free motion (45% of the time spent vs. 52% of the time spent for wt KOS (Figure 4A, right)) suggesting that MLD may be especially important for free diffusion. This is also in line with data displaying the behavior of the first segment upon landing, which show a larger fraction of free motion together with a smaller fraction of immobile particles for the wt KOS viruses (Figure 4B). The diffusion coefficient (Figure S7D) and the confinement radius (Figure S7E) of the first segments showed similar trends for all segments.

Taken together, these results indicate that the MLD promotes mobility of the virus on GAGs found at the cell surface by enhancing viral diffusion. The importance of this diffusion behavior in accelerating virus entry could be further appraised through virus entry experiments.

### 3.5. Deletion of the MLD on Glycoprotein C Delays Virus Entry

**Figure 5:**
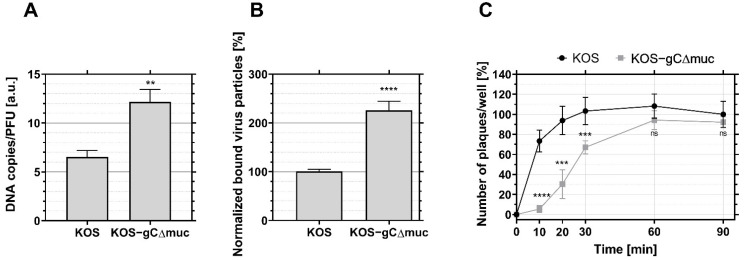
Influence of the mucin-like (MLD) domain of the glycoprotein gC on HSV-1 binding and entry kinetics into HaCaT cells. (**A**) DNA copies/PFU ratio for KOS and KOS-gCΔmuc. (**B**) Binding of wild type (KOS) and MLD-deleted (KOS-gCΔmuc) HSV-1 to HaCaT cells at the same concentration of infectious particles. The total number of particles bound in terms of DNA copies was estimated by qPCR. (**C**) Wild-type (KOS) and MLD-deleted (KOS-gCΔmuc) were added to HaCaT cells at equal concentrations of infectious particles (200 PFU/mL) and entry quantified at differenttime points. The number of plaques was normalized to the value for KOS at 90 min. Student’s *t*-test was used to assess the significance, ns = not significant, ** *p* ≤ 0.01, *** *p* ≤ 0.001 and **** *p* ≤ 0.0001 relative to the control HaCaT cells.

To confirm that the influence of the MLD on viral interactions with CS and HS is relevant to viral uptake into the target cell, we also characterized the entry efficiency for the mutant KOS-gCΔmuc and compared it to the wt virus KOS (Figure 5). Both virus preparations were characterized by distinct particle infectivities (in terms of DNA copies)/PFU ratios, with KOS-gCΔmuc exhibiting ~1.9 times higher particle/PFU ratios than the wt KOS (Figure 5A), which is consistent with our previous results [20] and also in line with the idea that the MLD promotes productive infection. As our primary aim was to investigate virus entry for productive infection, all experiments were carried out by adding the same concentration of infectious particles (i.e., the same PFUs). The quantification of virus particle binding by qPCR, revealed that ~2.2 more genome copies were bound to the surface of HaCaT cells for KOS-gCΔmuc than that of KOS-WT (Figure 5B). This difference closely reflects the difference in DNA copy/PFU ratios (Figure 5A) for both samples, and thus suggest that deletion of the MLD does not impair the ability to establish a firm contact with target cells.

An investigation of virus entry revealed that internalization of the mutant is delayed as compared to the wt virus, although, after 90 min, similar infection levels were reached for both viruses (Figure 5C): after 10 min, only 5–6% of the total KOS-gCΔmuc had entered the cells, while, for wt KOS, this amount reached 72%. This delay is unlikely to be caused by differences in the number of infectious virus particles that initially attach to the cell since: (i) under our experimental conditions, lower levels of virus binding are expected to lead to a reduction in the plateau value of the entry kinetics, rather than in the lag time in the entry kinetics; (ii) we report a higher number of virus particles being bound (in terms of DNA copies) for the mutant as compared to the wild type (Figure 5B).

The biophysical and biological results obtained here support the notion that the MLD of HSV-1 gC is a key regulator of early viral GAG interactions and subsequent navigation through the target cell glycocalyx, thereby accelerating cell penetration.

## 4. Discussion

Virus recruitment to the cell surface is a complex multistep process involving multivalent interactions with cell-surface ligands, including GAGs. We previously observed that HSV-1 virus particles binding to GAGs immobilized onto a substrate in a biomimetic fashion can diffuse laterally, presumably through the formation and dissolution of single bonds between viral glycoproteins and neighboring GAGs on the surface [16]. Thus, we hypothesized that the transport of the virus through the glycocalyx, as well as on the GAGs covering the cell membrane, could be associated with virus translocation from binding site to binding site. To further explore this process, we investigated the diffusive behavior of HSV-1 at the cell surface and its dependence on the presence of GAGs. Recent studies have shown that the mobility of the virus at the cell membrane is essential for virus entry [2,3,67], and while processes such as the cytoskeleton-driven surfing of HSV-1 on filopodia have been observed [68], very limited data have been found on viral diffusion during GAG-mediated initial recruitment at the cell surface and its effect on subsequent entry.

For our investigations, we developed an SPT method using high-speed fluorescence microscopy to track the initial binding of the virus at the cell surface. Dynamic data were analyzed by reconstructing virus trajectories with high resolution, followed by segmentation according to motion type. The moment scaling spectrum (MSS) was used to classify each segment type into immobile, restricted confined behavior, Brownian free motion or directed motion. MSS is a robust analytical method to identify the type of motion by using multiple-order moments in the displacement distribution, thus providing a more accurate diffusive behavior, which is needed for the SPT performed on live cell surfaces. Two cell models were used in our study: CHO-K1 cells, which lack entry receptors for HSV-1, and HaCaT cells that are permissive to HSV-1 infection. While these cells are unlikely to produce a thick glycocalyx in vitro, they are surrounded by a cell-membrane proximal glycocalyx, mostly leading to their two-dimensional mobility at the cell surface. Nevertheless, the surface of such cells represents an excellent model to study the influence of different GAGs or the MLD on GAG-mediated virus diffusion processes.

Here, we report a highly heterogeneous HSV-1 diffusion behavior upon landing on GAG-expressing CHO and HaCaT cells. Indeed, in the first minutes of virus–membrane interaction, the virus switches several times between being immobile, having confined diffusion, and free Brownian motion, suggesting that diffusion in different motion types may be needed to achieve efficient transport at the cell surface. Generally, the very first segment upon landing could be one of the three motion types, although the probability of free diffusion was slightly higher with higher diffusion coefficients than it was for the later segments. This observation may be related to the fact that CS chains, which were found to promote free diffusion, are longer than HS (20–70 kDa for CS [69] vs. 15–20 kDa for HS [12]), making it likely that they protrude further away from the cell surface, thereby increasing the probability of first encountering viruses.

The diffusion behavior reported in this work is likely to originate both from virus translocation along different binding sites on GAG chains [16], as well as from the lateral mobility of the GAG-bearing membrane-anchored proteoglycans in the cell membrane. Transmembrane proteins have been reported to exhibit either an active motion at the cell surface, if their movement is driven by intracellular motor proteins [17], or confined diffusion, due to membrane crowding and compartmentalization, e.g., by the cytoskeleton or through the formation of lipid domains [70]. Active motion was only occasionally observed in our experiments, indicating that transport through molecular motors does not significantly influence the initial GAG-mediated recruitment of HSV-1 at the cell surface. However, it could be hypothesized that, in our study, viruses exhibiting a confined diffusion behavior predominantly diffuse through anchoring on mobile membrane components, while the free-diffusing ones may hop from GAG to GAG to a greater extent. The latter hypothesis is supported by our previous observation that the diffusion of HSV-1 on mostly immobile, surface-bound GAGs, was predominantly free [16,31] as well as by previous studies indicating that the lateral movement of influenza A virus on immobile receptors is also governed by free diffusion [13]. However, it should be noted that studies on smaller particles such as the GAG-binding proteins have also reported that protein diffusion on fixed cells (abolishing the diffusion of the proteoglycan core) is associated with confined motion [17]. This effect has been attributed to a localized translocation behavior from binding site to binding site along the same or a neighboring HS, consistent with the view that the glycocalyx has a heterogeneous structure, where available protein binding sites are distributed heterogeneously in the glycocalyx matrix, forming local networks and paths. This highlights that the unambiguous linking of one type of diffusion to a particular mechanism is not straightforward. 

Next, the influence of the GAG type on virus particle diffusion was investigated. While the importance of both HS and CS in recruiting HSV-1 at the cell surface is well-documented [22,23], the distinct role played by each GAG in the process is unknown. We have reported an influence of the GAG type on HSV-1 diffusion using biomimetic GAG surfaces [16], and here we provide evidence that CS and HS have important but competing roles in modulating the mobility of the virus on live CHO-K1 cells: while CS enhances free motion with faster diffusion, HS mediates a restricted confined behavior with slower diffusion and higher confinement.

The diffusive hopping behavior of a virus on GAGs, which is expected to be mediated by the formation and dissolution of individual glycoprotein–GAG bonds, is likely to be determined by the affinity and kinetics of the individual monovalent interactions occurring between GAG chains and the viral glycoprotein gC, the main viral interaction partner of GAGs [23]. A fast exchange rate (high k_on_ and high k_off_) is likely to benefit viral diffusion. Indeed, studies on the fibroblast growth factors (FGF) interacting with HS and CS have shown that different binding affinities [71] are related to different diffusive behaviors [17]. A closer look at the characteristics of the individual virus–GAG bonds shows that, on the viral glycoprotein side, gC–GAG interactions occur via a well-defined GAG-binding domain located within the N-terminal part of the protein, which widely overlaps with CS- and HS-binding. This domain comprises clusters of hydrophobic and positively charged amino acids [23,26,66], where the charged amino acids can bind to negatively charged sulfate/carboxyl groups on the GAG chain. Relatively non-specific electrostatic interactions thus play an important role, although GAG recognition is dictated by a high degree of specificity. This is confirmed by the fact that the amino acid sequence of the GAG-binding site is well-conserved, and even small mutational changes in this part of the gC severely impairs the binding [23,55]. On the cell surface, it is likely that gC is not monospecific for one particular HS or CS structure, but is associated with more or less selective binding to several GAG structures. Thus, the conserved structure of the GAG binding site appears to be optimized to bind several HS or CS variants, representing binding structures present on each of the many tissue types targeted by HSV-1 [11].

Mårdberg et al. investigated the interaction of gC with CS and HS [23] and reported that the gC–CS interaction had a lower affinity than the HS counterpart (K_D_ ~2× higher on CS), while being more dependent on ionic interactions. This, together with our data, suggests that a weaker and possibly less specific gC–GAG interaction facilitates free diffusion and limits virus confinement on the cell surface. While the affinity of the individual virus-GAG bond is a good candidate to interpret the diffusive behavior, it is also worth considering that the overall avidity between the virus particle and cell-surface GAGs may influence both confined and free diffusion. Avidity depends not only on the affinity of the individual GAG–glycoprotein interactions but also on the number of bonds that are formed. In turn, the number of bonds that are formed is likely to depend (i) on the affinity of the monovalent interaction, where more bonds are likely to form for stronger monovalent interactions and (ii) on the density of the cell surface receptor in question [3]. In this context, a weaker gC–CS interaction [23] and the 3–10 times lower number of CS saccharides present on the CHO cell surface [72,73,74] are also in line with a scenario where the virus diffuses in a confined behavior on the cell surface by binding to multiple mobile membrane-embedded proteoglycans. In this case, binding to HS leads to a slower and more confined diffusion as compared to CS, which can be explained by the formation of more virus–proteoglycan bonds to HS compared to CS-carrying proteins.

Generally, a more dynamic interaction facilitating the detachment and reattachment of the virus particle may also enhance diffusive processes at the cell surface. In a previous study, where bilayers made of plasma membrane extracts were treated with heparinase, we reported a decrease in the association rate to 60%, as well as a ~2.5-fold increase in the dissociation rate [21], suggesting that the removal of HS influenced the binding and release of the virus to the cell surface. HS removal also resulted in a more mobile interaction [21], in agreement with our findings that HSV-1 has a more mobile and free motion on HS-deficient cells. Another study showed that HS expression increased during the initial stages of HSV-1 infection, which enhanced virus attachment to cells [19] and that, during the egress of HSV-1 from human corneal epithelial cells, the virus particles’ release was facilitated by the removal of HS via the increase in cellular enzyme heparanase [29,75]. It is worth highlighting that HSV-1 particles can be mobile at the surface of CHO and HaCaT cells without the cooperation of heparanase during the recruitment and early attachment prior to infection, and that the interaction with HS and CS is enough to create mobility. In light of our findings, this increase in HS concentration during virus recruitment can promote more confined behavior, as well as slower transport, which could reinforce efficient binding and subsequent transfer to the entry receptor(s), while the cleavage of HS with cellular heparanase during egress weakens the virus–GAG interaction, promoting free behavior that may facilitate virus release. Thus, the complexity of the cellular glycocalyx is an important factor influencing virus diffusion.

The cellular glycocalyx is a self-organized matrix characterized by a high degree of heterogeneity in space and time. First, enzymatic processes determine the biochemical identity of the GAGs by regulating the presentation of sulfated sugar sequences on the GAG chains. Second, the GAG-binding proteome of the cell adds another level of regulation by potentially occupying virus-binding sites though proteins of higher affinity. Thus, a combination of the availability of sulfated structure and level of occupancy of GAG-binding sites determines the virus’ binding properties to the cell’s glycocalyx. Such variations in HS and CS structures among different tissues might be important to modulate the GAG-dependent diffusion profile and lead us to speculate that the interaction profile may be fine-tuned and optimized for entry in a tissue in a specific manner. Further, it is highly likely that subpopulations of CS and HS overlap regarding their functions in mediating virus diffusion. As an example, we previously found that the highly sulfated CS-E motif has similar binding characteristics to HS. In particular, it may form efficient receptors for HSV-1, thus taking over the function of 3O-sulfated heparan sulfate as an entry receptor [53].

The differential role of GAGs in modulating virus diffusion at the cell surface is particularly interesting when considering the molecular organization of the glycocalyx where glycosaminoglycans favoring a fast and free diffusion are found further from the cell and secreted in the extracellular matrix, while the proteoglycans favoring confined motion are proximal to the cell surface. Indeed, in this scenario, the virus may take advantage of such an architecture to first successfully navigate the outer layer via rapid free diffusion before reaching proteoglycans that restrict their motion on the cell surface, which, through confined and more limited diffusion, may play a key role in directing and transporting the virus to the appropriate entry receptor(s). Such a figure could not be investigated in this study, as common cell culture systems, including CHO-K1 and HaCaT cells, do not express an extended glycocalyx or the extracellular matrix [76,77]; this aspect should be addressed by studying virus diffusion in more complex in vitro glycocalyx models or by using in vitro tissue models.

The GAGs’ role in the binding and entry of HSV-1 is further highlighted, in particular the significant role that HS plays in promoting viral entry. HS removal not only leads to a decrease in the number of virus particles attaching to the cell surface but also to a reduction in the fraction of particles that successfully infect the cell. We can speculate that this active role is associated with an optimized HS-mediated virus diffusion behavior at the cell surface, which is also in line with the previously reported contribution of 3-O HS to the receptor function for the pre-entry step mediated by gD [62]. Our data also reveal the differential role that CS plays in binding and entry. Indeed, the presence of CS is not essential for viral entry, at least not on HaCaT cells. Rather, the removal of CS led to an enhancement of virus attachment to HaCaT cells, in line with the idea that CS, which is hypothesized to be the first contact point of the virus, may delay or shield HSV-1 interactions with HS. This finding may illustrate the price, in terms of binding efficiency, that the approaching virus must pay to successfully navigate the glycocalyx.

Many enveloped GAG-binding viruses have been reported to be equipped with prominent clusters of O-glycans forming the MLD of the glycoprotein, suggesting that virus–membrane interactions may be modulated by an interplay of the effect of the cellular GAG profile and viral glycoprotein O-glycosylation. In this context, we have recently reported that, while not directly involved in GAG binding [78], the MLD affects the kinetics and affinity of the gC-CS interaction by facilitating dissociation, while, at the same time, increasing the affinity (K_D_ decreases by a factor ~10), presumably due to the higher on rate (higher k_on_) [20]. The presence of the MLD also increases the affinity (lower K_D_) of the gC-HS bonds by a factor of ~7.5, albeit mostly due to their higher on rate (higher k_on)_ [78]. Here, we further confirm the biological significance of this domain in the context of optimizing virus behavior in the glycocalyx and demonstrate that this structure greatly facilitates virus diffusion at the cell surface. The finding that the viral MLD not only facilitates diffusion at the cell surface but also accelerates viral entry is an observation that is likely associated with optimized interaction characteristics at the cell surface, including the particles’ more efficient transport to the receptors. These results are in agreement with our previous work on CS biomimetic systems [31], where we showed a decrease in the mobility and diffusion coefficient of the mutant HSV-1, which lacks MLD on gC. We previously reported that the MLD facilitates the dissolution of gC-GAG bonds [20,31], which is likely to facilitate free diffusion by virus translocation from binding site to binding site. A similar effect of the MLD was found on the related virus HSV-2, where deletion of the MLD decreased the mobility of the virions on CS [63]. On a molecular level, we hypothesize that the remarkable ability of the MLD to regulate virus diffusion in the glycocalyx may be associated with a tightly modulated electrostatic and steric repulsion between negatively charged glycans on gC molecules in the virus particle and the negatively charged GAGs. This idea is corroborated by the fact that the MLD stabilizes an extended rod-like confirmation of gC-1 [79] and that it is rich in negatively charged sialylated O-linked glycans. This is further supported by our previous studies on isolated gC and GAG molecules [20], showing that i) the MLD significantly restricts the number of gC copies that can bind per CS chain, likely regulating multivalency, and ii) that the removal of negatively charged sialic acids from the MLD on gC increases gC’s interaction with GAGs. This highlights MLD’s unique ability to act as a regulatory component, optimizing the binding characteristics at the cell surface in order to, among others, facilitate diffusion through the glycocalyx.

## 5. Conclusions

This study provides new key insights into infection mechanisms, with a focus on how viral and cellular factors regulate the dynamics of GAG-binding viruses at the cell surface, and how such processes determine entry efficiency. High-speed SPT using fluorescence microscopy allowed us to investigate the diffusive behavior of HSV-1 on live cells and its dependence on cell-surface GAGs and on the MLD of the viral attachment protein gC. A thorough analysis of the diffusion behavior reveals that different types of GAGs have distinct functions in modulating the dynamic behavior of viruses in the glycocalyx and that protein glycosylation, in the form of MLDs, can further contribute to this. As we suppose that this diffusive behavior is a consequence of the multivalent interactions between individual molecules, further investigations of individual glycoprotein–GAG bonds, using, for example, single-bond force spectroscopy, are needed. Combining our dynamic study with a static study of the interaction will make it possible to further elucidate the processes underlying the virus diffusion behavior reported here, and eventually shed light on the GAG-dependent mechanisms mediating the initial recruitment of the virus to the glycocalyx and cell surface, and the subsequent molecular hand-over to the pre-entry and entry steps. Moreover, given the ubiquitous MLD presence on GAG-binding viruses, the mechanisms revealed by our findings may govern how many viruses, from the respiratory syncytial virus to Ebola, are directed along the cell surface to their point of entry.

## Data Availability

Movies can be found through the figshare data repository private link: https://figshare.com/s/bb931529e56e6f909025, accessed on 8 July 2022.

## Supplementary Materials

The following supporting information can be downloaded at: https://www.mdpi.com/article/10.3390/v14081836/s1, Figure S1: Percentage of internalized viruses 5 min after viruses are added on top of CHO and HaCaT cell surface; Figure S2: The switch rate of viral particles between immobile, confined and free motion types; Figure S3: Characterization of the GAG levels CHO cells by immunofluroscence; Figure S4: Duration of all segments for the three motion types; Figure S5: Influence of the nature of the glycosaminoglycans on the first segment of HSV-1 (KOS) interaction with the CHO cell surface; Figure S6: Influence of the nature of the glycosaminoglycans on the initial interaction of HSV-1 with the HaCaT cell surface; Figure S7: Influence of the mucin-like domain on the first segment of HSV-1 (KOS) interaction with the HaCaT cell surface; Figure S8: Linear regression analysis for binding quantification via qPCR; Figure S9: Number of attached HSV-1 virus particles at the CHO cell surface; Movie S1: Representative movie for each condition.
