# Supplementary material for: Cellular Chondroitin Sulfate and the Mucin-like Domain of Viral Glycoprotein C Promote Diffusion of Herpes Simplex Virus 1 While Heparan Sulfate Restricts Mobility
Source: Viruses. 2022 Aug 21;14(8):1836. doi: 10.3390/v14081836 (PMC9412521; doi:10.3390/v14081836)
Supplement: Supplementary file 1 [file viruses-14-01836-s001.zip › viruses-1835823-supplementary.pdf]

Supplementary material for

Cellular Chondroitin Sulfate and the Mucin-like Domain of Viral Glycoprotein C Promote Diffusion of Herpes Simplex Virus 1 while Heparan Sulfate Restricts Mobility

Yara Abidine[1-2+], Lifeng Liu[1-2+], Oskar Wallén[1], Edward Trybala[3], Sigvard Olofsson[3], Tomas Bergström[3], Marta Bally[1-2*]

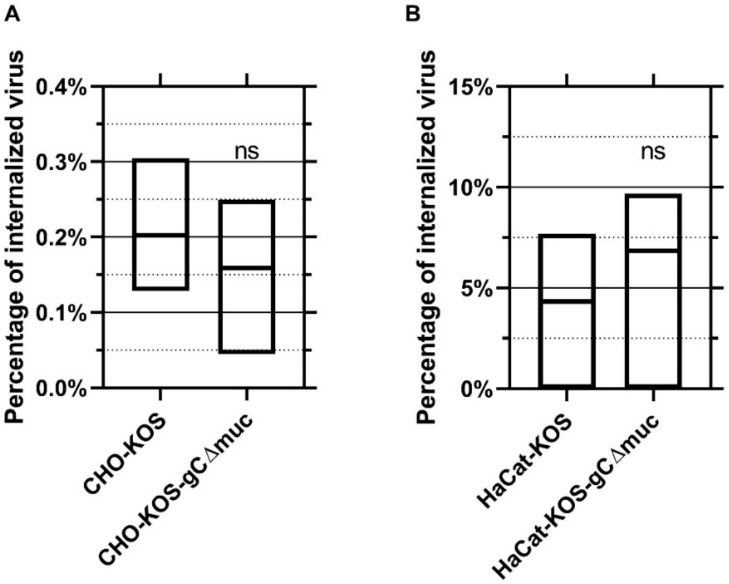

Figure S1. Percentage of internalized viruses 5 minutes after viruses are added on top of CHO and HaCaT cell surface. The percentage of intracellular KOS and KOS-gCΔmuc was quantified by binding the viruses to the CHO (**A**) and HaCaT (**B**) cell surfaces for 5 min followed by quenching of the extracellular labelled viruses with trypan blue. Briefly, the labelled virus particles were added on CHO or HaCaT cells in similar way as done for an SPT experiment. After 5 min, 10 µl of trypan blue (T8154, Sigma) was added to the well on top of the microscope. Positions before and after quenching were saved and imaged using 60X oil immersion objective and 405-488 nm filters and differential interference contrast (DIC). For each position, the number of viruses before and after quenching was quantified using multi-point tool in ImageJ and the ratio of intracellular/before quenching was calculated. Asterisks denote a significant difference by Welch t-test: ns=not significant, *p<0.05, **p<0.01, ***p<0.001 and ****p<0.0001 relative to KOS.

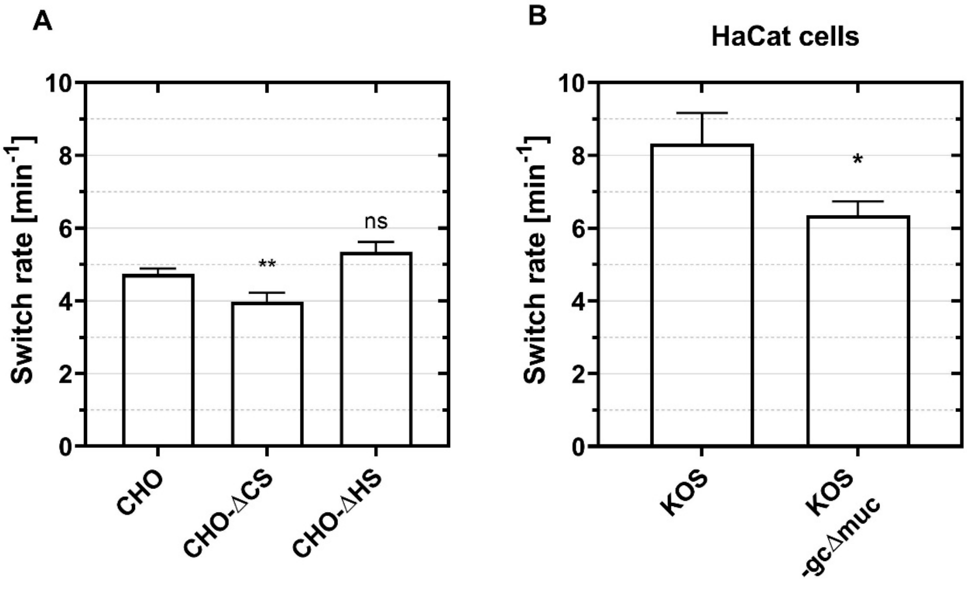

Figure S2. The switch rate of viral particles between immobile, confined and free motion types. The switch rate represents the number of segments per minute for each condition. (**A**) HSV-1 (KOS strain) on cell surfaces with different glycosaminoglycan composition: CHO-K1 cells (CHO) expressing both heparan sulfate (HS) and chondroitin sulfate (CS), CHO treated with Chondroitinase ABC (CHO-ΔCS) and pgsD-677 lacking HS (CHO-ΔHS). (**B**) On HaCaT cells, the switch rate of HSV-1 (KOS strain) and the HSV-1 lacking the mucin-like domain (KOS-gcΔmuc) was measured. Asterisks denote a significant difference by Welch t-test: ns=not significant, *p<0.05, **p<0.01, ***p<0.001 and ****p<0.0001 relative to control.

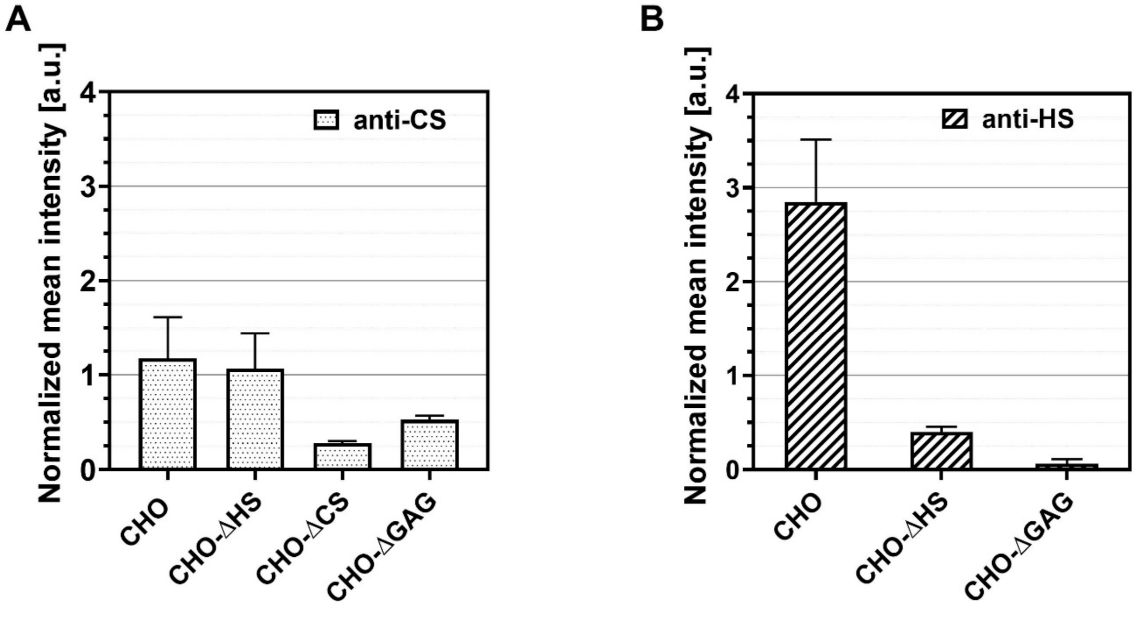

Figure S3. **Characterization of the GAG levels CHO cells by immunofluroscence.** (**A**) Anti-chondroitin sulfate (anti-CS) and (**B**) anti-heparan sulfate (anti-HS) antibodies were used with AlexaFluor 488 secondary antibody to characterize the surface of CHO-K1 cells expressing both HS and CS (CHO), CHO treated with Chondroitinase ABC (CHO-ΔCS), pgsD-677 lacking HS (CHO-ΔHS) and pgsA-745 lacking GAGs (CHO-ΔGAG). The data is normalized by subtracting the background and dividing by the value of the control (secondary antibody only). The bars represent standard error of the mean (SEM) and every condition has a minimum of n=10. A student t-test was used to compared antibody intensites after enzymatic treatment leading to following p-values when comparing to untreated cells in panel (A): non-significant for CHO-ΔHS and **$p<0.001$ for CHO-ΔCS

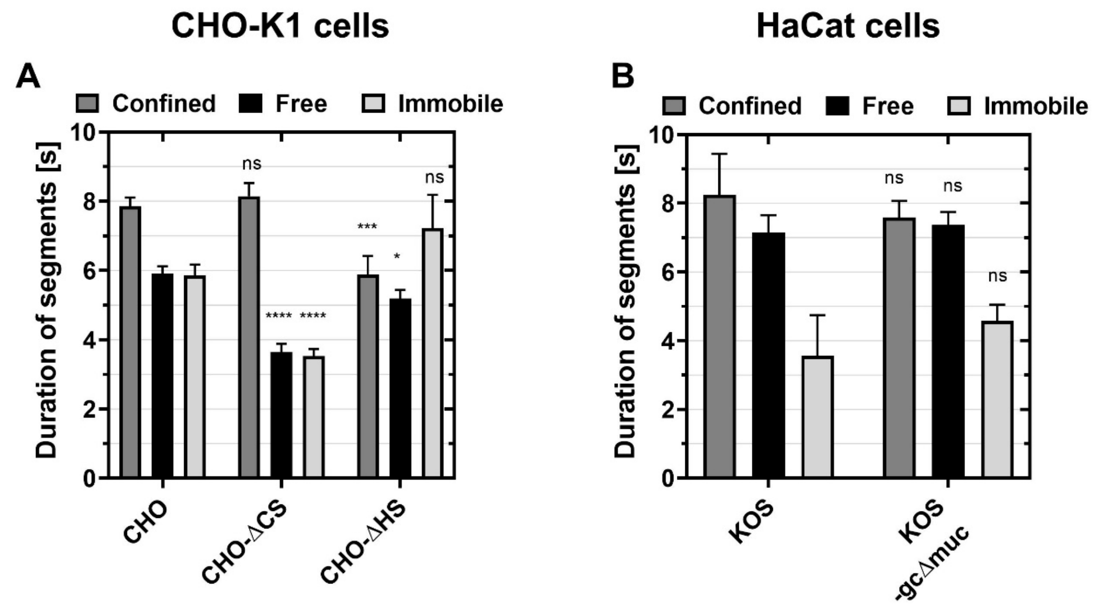

Figure S4. Duration of all segments for the three motion types. The motion types are immobile (light grey), confined (grey) and free (black). (**A**) The mean duration of segments for CHO-K1 cells expressing both heparan sulfate (HS) and chondroitin sulfate (CS) (CHO), CHO treated with Chondroitinase ABC (CHO-ΔCS) and pgsD-677 lacking HS (CHO-ΔHS). (**B**) On HaCaT wt cells: the mean duration for HSV-1 (KOS strain) and the mutant HSV-1 lacking the mucin-like domain (KOS-gCΔmuc). The error bars represent standard error of the mean (SEM) with a number of values ranging between 132 and 1281 depending on the condition. Asterisks denote a significant difference by Welch t-test: ns=not significant, *$p<0.05$, **$p<0.01$, ***$p<0.001$ and ****$p<0.0001$ relative to CHO cells (A) or wt KOS on HaCaT (B).

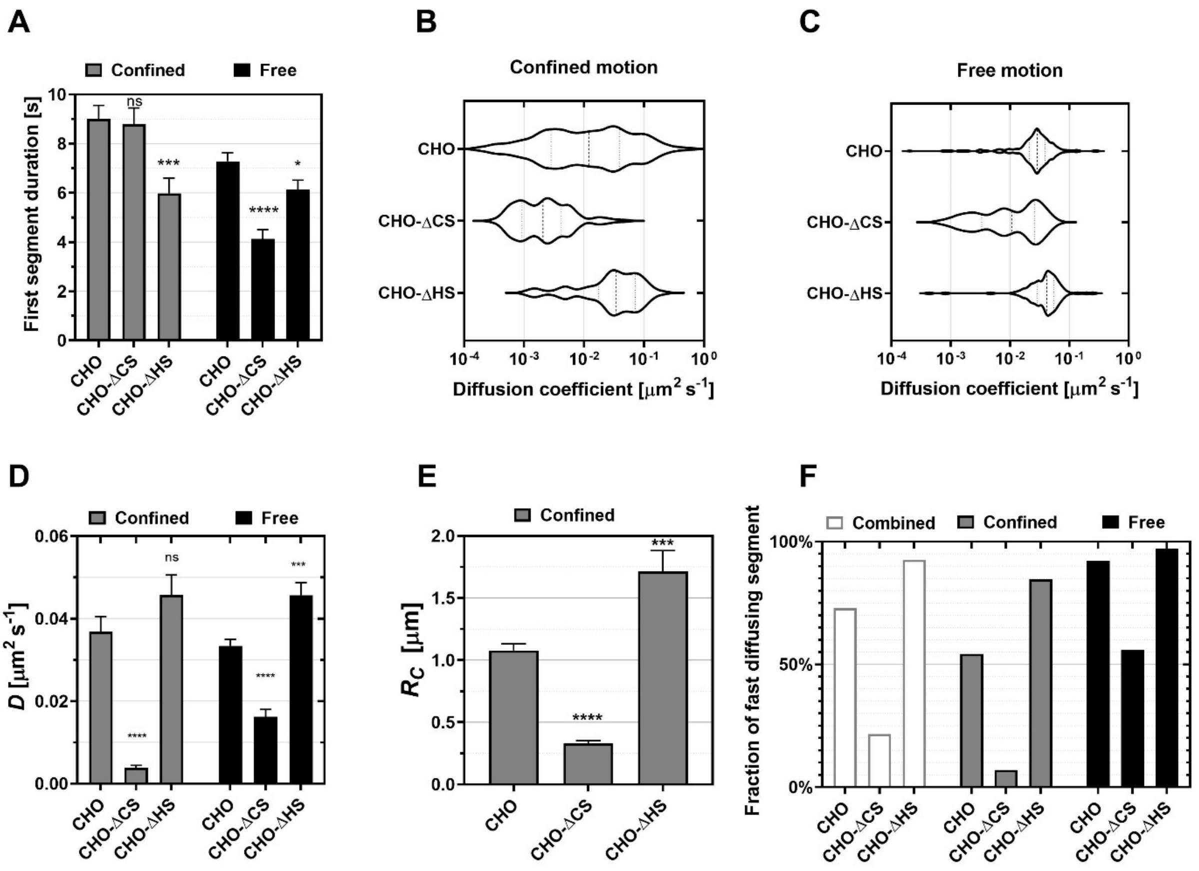

Figure S5. Influence of the nature of the glycosaminoglycans on the first segment of HSV-1 (KOS) interaction with the CHO cell surface. The cells surfaces are CHO-K1 cells expressing both HS and CS (CHO), CHO treated with ChABC (CHO-ΔCS) and pgsD-677 lacking HS (CHO-ΔHS). (**A**) The duration of the first segment, in the confined (grey), free (black) motion type for each condition. (**B-C**) Distributions of the diffusion coefficients of the first segment plotted in violin plots show two populations: slow and fast particles classified according to a cutoff of 10^{-2} $\mu m^2/s$. (**D**) The mean of the diffusion coefficient of the full distribution presented in C. (**E**) The associated confinement radius of the confined motion. (**F**) The fraction of particles exhibiting fast diffusion are presented for the confined and free diffusion combined (white) and each motion type separated. The total number of viruses used here is 644, 204 and 176 for CHO, CHO-ΔCS, CHO-ΔHS respectively. Data was acquired in at least three independent experiments. Asterisks denote a significant difference by Welch t-test: ns=not significant, *p<0.05, **p<0.01, ***p<0.001 and ****p<0.0001 relative to control.

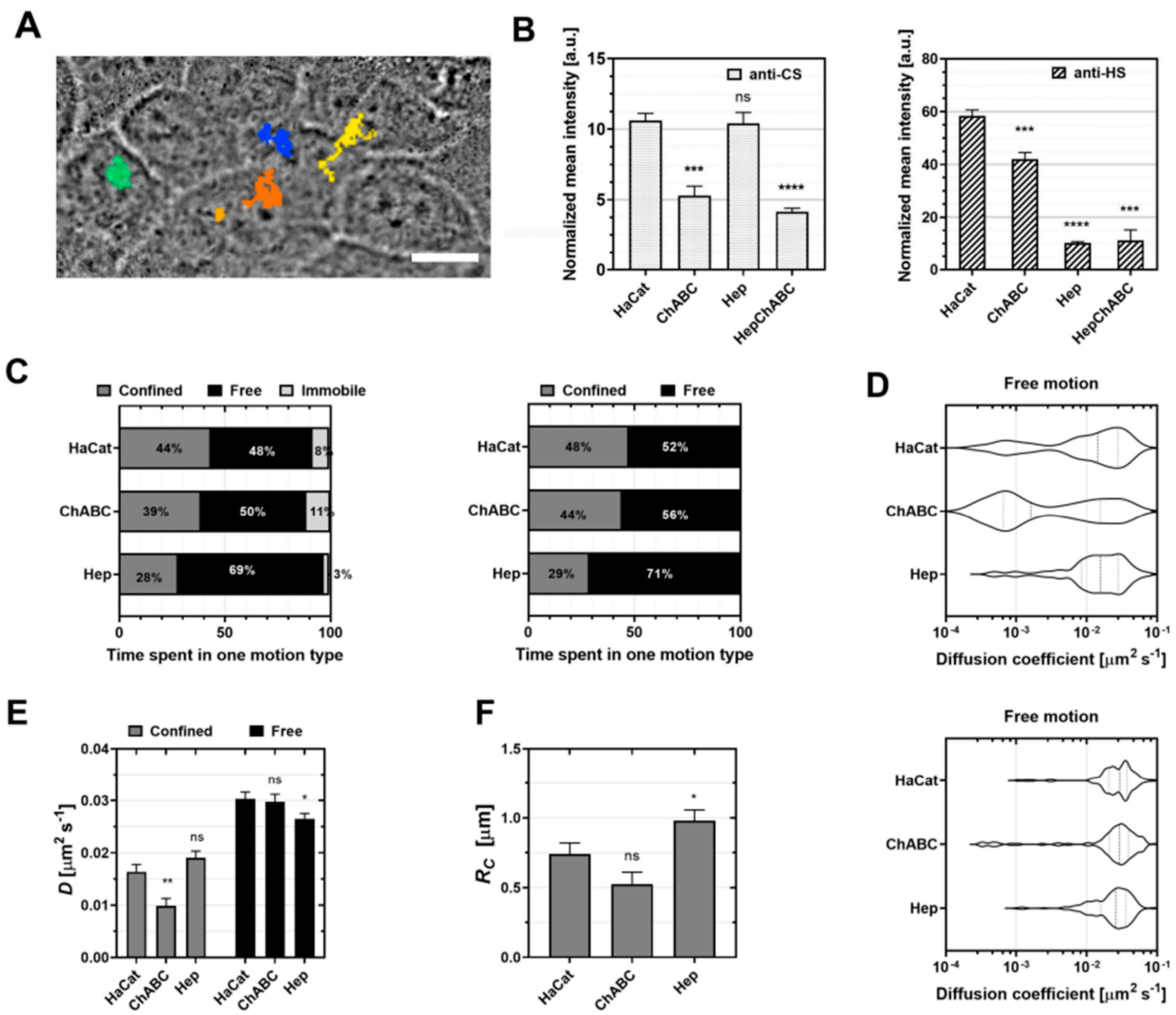

Figure S6. Influence of the nature of the glycosaminoglycans on the initial interaction of HSV-1 with the HaCaT cell surface. (**A**) Representative tracks of HSV-1 particle landing and diffusing at the HaCaT cell surface. The virus trajectory was recorded at 25 fps and was constructed using ImageJ. Bar is 10 μm (**B**) Anti-chondroitin sulfate (anti-CS) and anti-heparan sulfate (anti-HS) antibodies were used with AlexaFluor 488 secondary antibody to characterize the surface of HaCaT cells expressing both HS and CS, HaCaT cells treated with heparinase 1 and 3 (Hep), HaCat cells treated with chondroitinase ABC (ChABC), and HaCaT cells treated with both heparinase 1+3 and chondroitinase (HepChABC). The data is normalized by subtracting the background and dividing by the value of the control (secondary antibody only). The bars represent standard error of the mean (SEM) and every condition has a minimum of n=7. (**C**) The fraction of the total time spent by KOS particles in one of the motion types. Left: confined (grey), free (black) and immobile (light gray); Right: mobile particles only (confined (grey) and free (black). (**D**) Distributions of the diffusion coefficients of the first segment plotted in violin plots. (**E**) The mean of the diffusion coefficient of the full distribution presented in C. (F) The associated confinement radius of the confined motion. Data was acquired in at least three independent experiments. Asterisks denote a significant difference by Welch t-test: ns=not significant, *p<0.05, **p<0.01, ***p<0.001 and ****p<0.0001 relative to control.

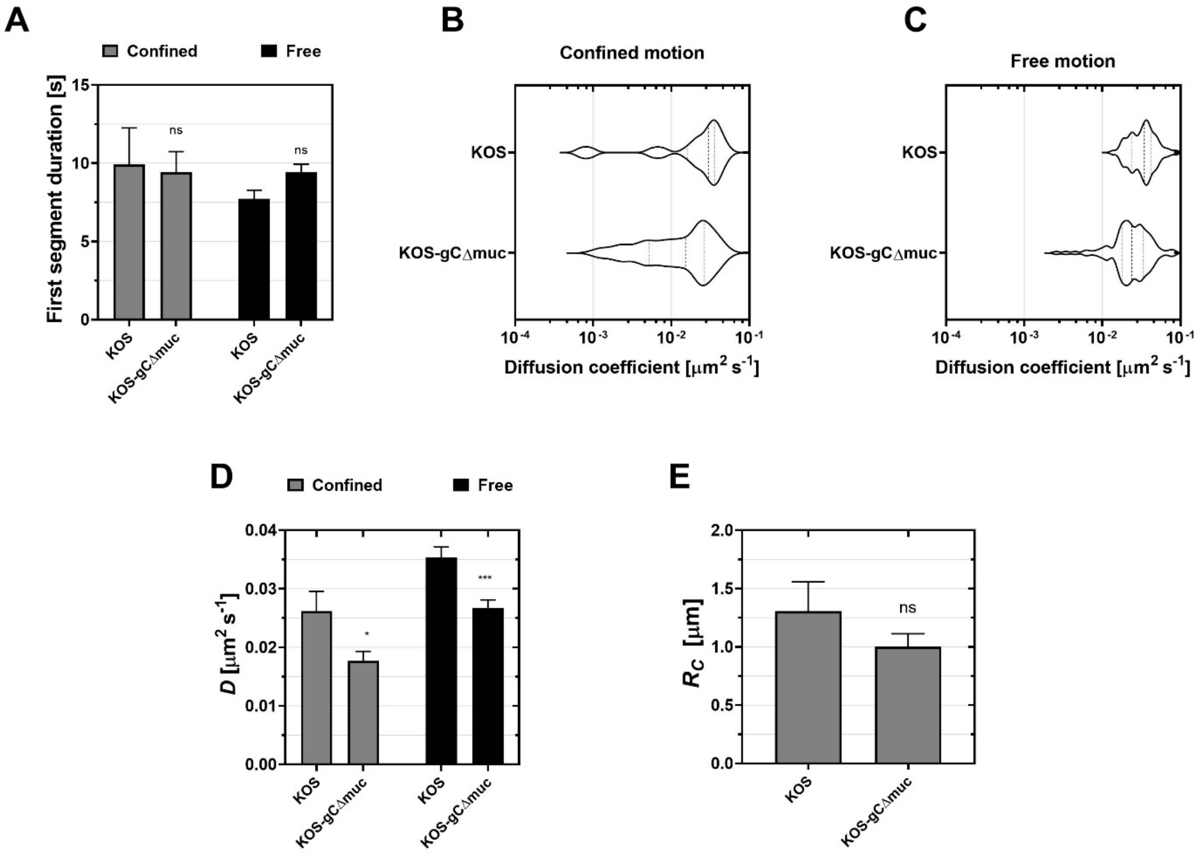

Figure S7. Influence of the mucin-like domain on the first segment of HSV-1 (KOS) interaction with the HaCaT cell surface. (**A**) The duration of the first segment, in the confined (grey), free (black) motion type for the two HSV-1 strains. (**B-C**) Distributions of the diffusion coefficients of the confined and free motions of the first segment plotted in violin plots. (**D**) The mean of the diffusion coefficient of the full distribution presented in B-C. (**E**) The associated confinement radius of the confined motion. The total number of viruses used here is 80 and 201 for KOS and KOS-gCΔmuc respectively. Data was acquired in at least three independent experiments. Asterisks denote a significant difference by Welch t-test: ns=not significant, *$p<0.05$, **$p<0.01$, ***$p<0.001$ and ****$p<0.0001$ relative to control.

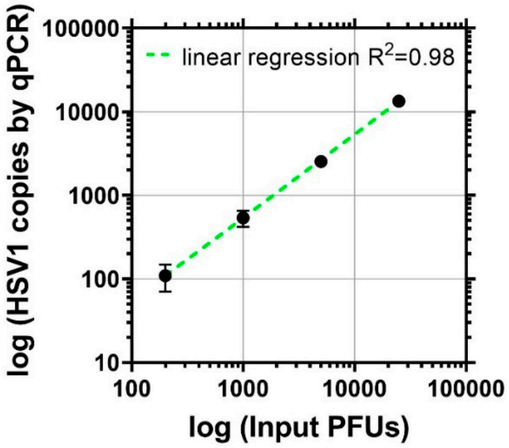

Figure S8. Linear regression analysis for binding quantification via qPCR. Binding quantification was done as described in method with different inputs, including 200, 1000, 5,000 and 25,000 PUFs. A linear regression was performed using the data from 4 repeats. Note: HSV1 copies by qPCR were only detected in two experiments with 200PFUs.

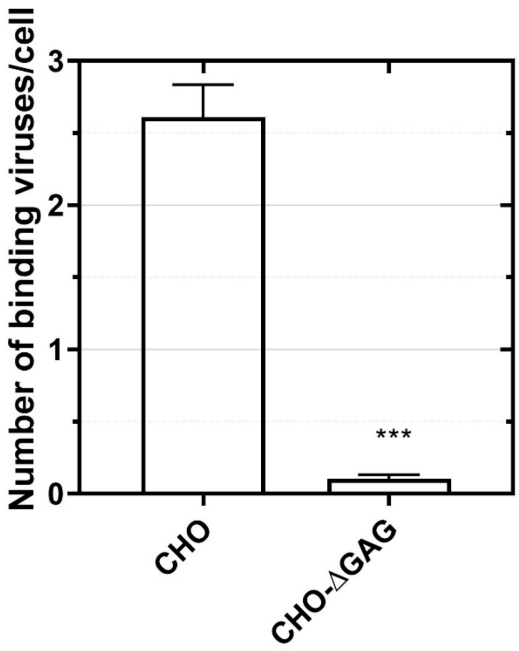

Figure S9. Number of attached HSV-1 virus particles at the CHO-K1 cell surface. Binding of the wild type HSV-1 (KOS strain) 10 minutes after adding the virus inoculum on CHO-K1 expressing both HS and CS and pgsA-745 lacking GAGs (CHO-ΔGAG). After a rinsing step to remove unbound particles, labelled particles and cells were counted using the multipoint tool in Fiji and the total number of viruses was divided by the number of cells. Each condition has at least 10 repeated measurements. A student t-test was used to assess the significance ***$p < 0.001$ relative to the wild type CHO cells.

Movie S1. **Representative movie for each condition**. Timelapses of HSV-1 (KOS strain) diffusing at the surface of (**A**) wt CHO-K1 cells expressing both herapan sulfate (HS) and chondroitin sulfate (CS), (**B**) wt CHO cells treated with chondroitinase ABC, lacking chondroitin sulfate (CHO-ΔCS), (**C**) pgsD-677 lacking HS (CHO-ΔHS). (**D**) Timelapse of HSV-1 (KOS strain) on wt HaCaT cells. (**E**) Timelapse of the mutant HSV-1 (KOS-gCΔmuc) on wt HaCaT cells. The scale bar represents 10 μm. Using Fiji, all movies were corrected for uneven background using the same procedure with a rolling ball of 50 and the noise was despeckled. The LUT thresholds were set to be 10 as minimum and 50 as maximum for all videos. Finally, the movies were then saved as .avi using an acceleration of 50 frame per second. (**F**) The virus trajectories reconstructed using Trackmate are shown on the movie corresponding to A for wt CHO. The colors of the trajectory are arbitrary.

Movies can be found through the figshare data repository private link: https://figshare.com/s/bb931529e56e6f909025

Raw data for Movies S1 A-E is available through figshare data repository private link: https://figshare.com/s/5b42786ce133f45983b6
